# Strong Correlation of *MTHFR* Gene Polymorphisms with Breast Cancer and its Prognostic Clinical Factors among Egyptian Females

**DOI:** 10.31557/APJCP.2021.22.2.617

**Published:** 2021-02

**Authors:** Moataza H. Omran, Basma E. Fotouh, Wafaa G. Shosha, Abeer Ismail, Noha E. Ibrahim, Shimaa S. Ramadan

**Affiliations:** 1 *Department of Microbial Biotechnology, Genetic Engineering Division, National Research Centre, Cairo, Egypt. *; 2 *Department of Chemistry, Faculty of Science, Helwan University, Cairo, Egypt. *; 3 *Department of Clinical and Chemical Pathology, National Cancer Institute, Cairo University, Giza, Egypt. *

**Keywords:** Methylenetetrahydrofolate reductase, C677T, A1298C, single gene polymorohisms (SNPs)

## Abstract

**Introduction:**

Globally, Breast cancer (BC) is considered the second most common type of cancer and the principal cause of death among affected women. Aim: In this study, we targeted to demonstrate the association of *MTHFR* single gene polymorphisms (*SNPs*) with the susceptibility of breast cancer, in addition to its correlation with the clinical patient features.

**Patients and Methods:**

This work was conducted on 100 Egyptian females with breast cancer and 60 healthy matched controls. Clinical examinations and pathological investigations were recorded. Genotyping of MTHFR polymorphisms *C677T* (rs1801133) and *A1298C* (rs1801131) by using Restriction Fragment length Polymorphisms (RFLP) and Sequencing assays were performed. Univariate, Multivariate and Haplotype analysis for the allelic frequencies and the association with clinicopathological features of BC were assessed.

**Results:**

The present data showed a strong significant association between the CT and TT of *MTHFR *(C677T), and AC and CC of (A1289C) with the susceptibility of BC showing highly statistical P- value (0.001). It was also demonstrated that the most frequent haplotype of the two loci of *MTHFR* (rs1801133-rs1801131) was TC. The latter was strongly associated with the aggressive clinical features of each of tumor size, advanced stage, involvement of cancer in lymph nodes, overexpression of *HER2neu* and dual negativity of both ER and PR hormones.

**Conclusions:**

SNPs within the *MTHFR* gene (C677T) and (A1289C) have strong correlation with BC among Egyptian females; These SNPs should be considered as important prognostic markers for identifying the individuals at high risk of developing BC and its progression.

## Introduction

 Worldwide, Breast cancer (BC) is considered a fundamental health problem and it is the principal cause of cancer death in females (Siegel et al., 2019). It is more common in developing countries. In Egypt, Carcinoma of the breast is the most prevalent cancer among Egyptian women representing about 35% of total cancer cases (Ibrahim et al., 2014). It has been shown that genetic factors interact with numerous environmental factors playing an important role in the pathogenesis of Breast cancer (Liu e al., 2016; Floris et al., 2020). Strong evidence by research revealed that folate metabolism imbalance plays a crucial role in cancer predisposition, based on its involvement in nucleic acid synthesis, methionine regeneration, oxidation and DNA synthesis (Carvalho et al., 2012). 

Folate metabolism is controlled by several polymorphic genes. Methylenetetrahydrofolate reductase (MTHFR) gene is one of these several genes located on chromosome 1 and is the prime form of folate in plasma giving the methyl group for methionine synthesis affecting DNA profiles by mediating the irreversible conversion of 5, 10-methylenetetrahydrofolate (5, 10-MTHF) to 5-methyltetrahydrofolate (5-MTHF) (crider et al, 2012; Castigilia et al., 2019). There are two functional and common single nucleotide polymorphisms (*SNPs*) in the *MTHFR* gene, *C677T* (rs 1801133) and* A1298C* (rs 1801131) that play a role in decreasing enzyme activity and increasing levels of plasma homo-cysteine (Castigilia et al., 2019; Floris et al., 2020). The *C677T* polymorphism takes place in exon 4, which involves a C to T substitution at position 677, leading to the substitution of the amino acid alanine to a valine at codon 222 (Ala222Val) in the N-terminal catalytic domain. While, the MTHFR A1298C polymorphism, takes place in exon 7.This polymorphism results in changing the glutamic acid to alanine at codon 429 (Glu429Ala) in the C-terminal regulatory domain of the protein (Weisberg et al., 2001; Waseem et al., 2016). Previous studies revealed the role of the interaction between these polymorphisms in the biosynthesis, repair and stability of DNA, causing abnormal gene expression, stimulating proto-oncogenes, over growth and aggressive cell transformation resulting in carcinogenesis (Kekeeva et al., 2006; Nazki et al., 2014). 

Breast cancer might be invasive that grows into the surrounding tissues or noninvasive that does not grow beyond the lobules or the ducts of the breast. Ductal carcinoma represents the majority of breast cancer where the cancer spreads in the cells lining milk ducts (Rakha et al., 2010; Gorringe et al., 2017). 

Breast cancer could be in early or advanced stages including stages I, II and III. These stages describe the cancer location, its extent of growth and spread. It might spread into the near lymph nodes or to distant parts of body as lungs, liver and bones( through blood and lymph vessels) that is called metastatic breast cancer (stage IV) (Veronesi et al., 2009; Donepudi et al., 2014). 

In few studies, the association of the single nucleotide polymorphisms with the main clincopathological features of breast cancer has been proposed (Karimian et al., 2020). These SNPs have also been correlated with the increasing risk of other cancers as cervical cancer, ovarian cancer, lung cancer, pancreas cancer, prostate cancer (Xia et al., 2014; Hajiesmaeil et al., 2016; Moghaddam et al., 2016; Zhong et al., 2019). 

Therefore, this work aims to study the association between *MTHFR* gene polymorphisms (*C677T* and *A1298C*) and the risk of developing breast cancer in Egyptian females. Furthermore, this is a prior study targets to clarify the linkage between the genetic basis of breast cancer and the appearance of aggressive clinical feature, providing a molecular basis for using these polymorphisms as markers for the modulation and the prognosis of breast cancer disease.

## Materials and Methods

*Subjects and Methods*

*Subjects (cases and controls)*

This study was conducted on 100 Egyptian females cases who had been clinically and histologically proven to be breast cancer and 60 healthy control Subjects. Patients’ clinicopathological data for each case were collected from their medical files at National Cancer Institute, Cairo University. The recorded information at diagnosis has the following inclusions: (1) age, (2) menopausal status, (3) family history, (4) Scarff-Bloom-Richardson (SBR) grade, (5) tumor size, (6) involvement of cancer in lymph node, (7) metastases, (8) histology type, (9) human epidermal growth factor receptor 2 (Her-2), (10) hormone receptor status including: estrogen receptor (ER) and progesterone receptor (PR). 

While, Exclusion criteria included: 1) Former history of any other types of cancer 2) Causes of infection by different viruses, 3) uncontrolled diabetes mellitus, 4) history of long-term drug use, 5) Auto-immune diseases. The healthy subjects were matched to cases with reference to ethnicity, age, gender, no history of acute or chronic diseases and absence of any infected viruses, or disorders. Before enrollment in the study written informed consent for research participation was signed from both pa¬tients and controls. The protocol of this study has the ethical approval no. 16472 as it was reviewed by the ethical committee of the National Research Center, Cairo, Egypt. This research was conducted in accordance with the principles of the Declaration of Helsinki.

*Methods*

Genomic DNA extraction: this was done from whole blood samples collected from 100 cases and 60 healthy controls by using Salting out method as described earlier (Omran and Fotouh et al., 2013). The quantity and quality of DNA were checked on a Nano Drop spectro-photometer and 0.8% (w/v) agarose gel electrophoresis, respectively. The DNA was stored at -80°C until further analysis. 

*Genotyping*

**Figure 1:**
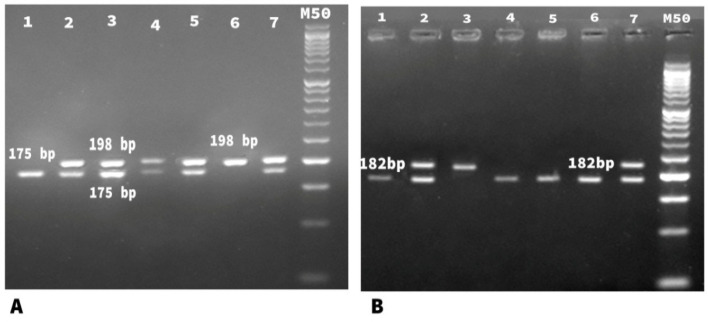
Agarose Gel Electrophoresis of Digested MTHFR C677T & MTHFR A1289C Amplicons (A) *MTHFR C677T*: Lane 1represents homozygous cut (TT) (175bp), Lanes 2,3,4,5,7 represent heterozygous cut (CT) (198bp & 175 bp), Lane 6 shows no cut (CC) (198bp), (M) represents 50bp molecular weight marker. (B) *MTHFR A1289C*: Lanes 1,4,5,6 show homozygous cut (CC) (182bp), Lanes 2,7 show heterozygous cut (AC) (237 bp and 182 bp), Lane 3 represents no cut (AA) .(M) represents 50bp molecular weight marker

Genomic variants of *MTHFR* polymorphisms (*C677T* and *A1298C*) were analyzed by (PCR-RFLP) assay. Amplification of MTHFR (C677T) was detected by forward primer 5′- TGA AGG AGA AGG TGT CTG CGG GA-3′ and reverse primer 5′-AGG ACG GTG CGG TGA GAG TG -3′. While, Amplification of MTHFR (A1298C) was performed using forward primer 5′-AAGGAGGAGCTGCTGAAGATG-3′and reverse Primer 5′-CTTTGCCATGTCCACAGCATG-3′. PCR Reaction mix with final volume (50 uL) contained 2 units Taq polymerase (finnzymes, Finland), 5 μLof 10X PCR buffer (supplied with the enzyme),0.2 mM dNTPs( Promega, Madison WI, The USA), 1.5 mMMgCl_2_, 5 μM from each primer, 200 ng of DNA and DDW to 50 μL. Thermal cycling protocol comprised: initial denaturation at 95˚C for 5 minutes, followed by 40 cycles each including 94˚C for 30 sec, 62˚C for 30 sec, and 72˚C for 30 sec, and a final extension step at 72˚C for 10 minutes .Success¬fully amplified products were 198bp of MTHFR ( C677T) and 237 of MTHFR (A1298C) were digested with 10 units of restriction endonucleases: Hinfl enzyme for ( C677T) and MboII enzyme for (A1298C) (Promega, Madison, WI, USA ) and incubated at 37° C overnight (Frosst et al., 1995; Cicek et al., 2004). Restricted fragments were resolved on 3% agarose gel electrophore¬s in parallel with a DNA size marker (Amersham Pharma¬cia- Biotech). The presence of (TT) genotype of C677T was indicated by complete digestion (homozygous cut) of the 198 bp PCR product using Hinfl enzyme into two fragments of (175 bp and 23 bp). While, (CC genotype) of A1289C was indicated by the complete digestion (homozygous cut) of the 237 bp PCR product using MboII enzyme into the fragments of (182 bp and 28 and 27 bp). However, the presence of (CC genotype) of C677T and (AA genotype) of A1289C was indicated by absence of each of Hinfl and MboII sites respectively generated an indigestible sequence (No-cut) (Figure 1).

**Figure 2:**
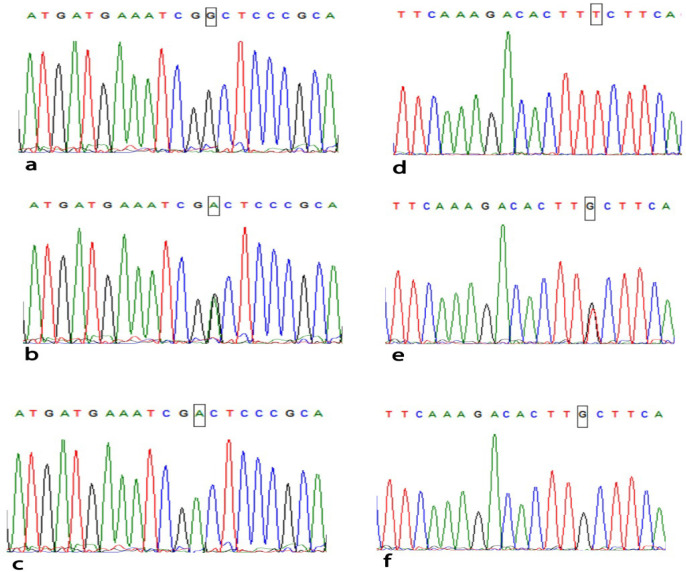
Sequencing Analysis Using Reverse Primer of *MTHFR C677T* & *A1298C* Genotypes. MTHFR C677T: (a) wild type CC genotype, (b) heterozygous CT genotype, (c) homozygous mutant TT genotype. MTHFR A1289C: (d) wild type AA genotype, (e) heterozygous AC genotype, (f) homozygous mutant CC genotype

Sequencing Analysis DNA sequence was performed using reverse primer to the PCR products of (CC, CT and TT) genotypes of (*C677T*) and (AA, AC, CC) genotypes of (A1298C) to confirm the *MTHFR* polymorphisms of *(C677T* and *A1298C*) (Figure 2). The sequenced samples were analyzed in the Au¬tomated Sequencer “ABI Prism 310 Genetic Analyzer”. The sequences were aligned with the consensus sequences that were retrieved from Gene Bank using the program ClustalX implemented in the Bioedit package (Omran and Nabil et al., 2013).

Statistical analysis was performed using statistical software SPSS (Statistical Package for Social Science) statistical program version (20.0) and Microsoft Excel program version 2016. Quantitative data were statistically represented in terms minimum, maximum, mean, standard division (SD) and median. Qualitative data were statistically represented in terms number and percent. Comparison between difference groups in the present study was done using Independent samples T-Test for comparing two parametric groups and by using Chi-Square Test with Relative Risk and Odds ratio, and the analysis of association between each polymorphism and breast cancer risk was presented in odds ratios (OR) with corresponding 95%. The allelic frequencies of each SNP were compared between cases and controls and the wide-type genotype was regarded as the reference group. Haplotype frequency distributions were deduced from genotype data and compared between cases and controls. The most common haplotype was selected as the reference. Odds ratios and 95% CI were calculated to estimate the degree of the association between haplotypes and the risk of breast cancer. A difference between groups was considered to be significant if P < 0.05 (Sole et al., 2006). 

## Results

*Clinicopathological Data of Patients with Breast Cancer*

Table 1, It shows all of the clinical and pathological parameters of BC patients enrolled in this study: age, family history, menopausal status, histological type, tumor size and stage, involvement of cancer in lymph node, metastases. It also shows hormone receptor status including: estrogen receptor (ER) and progesterone receptor (PR), human epidermal growth factor receptor 2 (HER2 neu). Most cases had mean age above 45 years (63%) and were post-menopausal (60%). Also, (66%) of patients were sporadic as they did not have a family history of BC. Histopathologically, most of patients (83%) had intermediate tumor grade (2). Moreover, a large proportion of them (80%) had invasive ductal carcinoma (IDC) and (53%) showed advanced stage tumor (III). Furthermore, most of these cases harbored a positive Her-2 neu (69%), ER- negative (54%) and PR- negative (58%). However, some of them (37%) and (27%) had involvement of cancer in lymph nodes N1 and N2 respectively, while metastasis was confirmed in only 4% of cases.

*Genotypes and alleles distribution of MTHFRC677T and MTHFRA1289C genes in patients with Breast Cancer and Controls*

Table 2, shows a strong significant association of the SNPs of C/T genotypes of *MTHFRC677T* and the A/C genotypes of *MTHFRA1289C* with BC in four genetic models including dominant, recessive, codominant, and overdominant models with highly significant P value (P=0.001). The percentage of CT and TT genotypes of C677T (55% and 27% respectively) was much higher in affected patients versus (20% and 0%) in control group. In the second study, it was demonstrated that the percentage of CC (46%) of MTHFRA1289C was much higher in patients versus controls (6.7%) with a highly significant P value (P=0.001). Moreover, it is observed that the patients with breast cancer had a significant association with the T allele of *MTHFRC677T* and with the C allele of* MTHFRA1289C* (P=0.001). Furthermore, it was found that the haplotype CT/AC followed by CT/CC, TT/AC and TT/CC was associated with elevated risk of BC (P=0.001).

*Statistical Analysis*

Table 3, clarifies the data that was analyzed by the Univariate statistical analysis that involved only one variable to enlighten patterns within the data. In two separate studies this analysis revealed strong significant correlation of the SNPs of (TC+TT) of *MTHFR C677T* in one study and that of (AC+CC) of *MTHFR A1298C* in the other study versus each of the BC clinicopathological data including: dual negativity of ER and PR, high HER2neu expression, involvement of cancer in lymph nodes and advanced tumor stage (III-IV) and bigger size (T2-T3) giving highly significant P values (0.001). 

Table 4, demonstrates the Multivariate statistical analysis by using a logistic regression that was done between three or more variables of the data in two separate studies evaluating the association of the SNPs of each of *MTHFR C677T* and those of *MTHFR A1298C* versus the BC clinicopathological data. This analysis discovered very highly significant P values (0.001) of (CC vs CT-TT) of *MTHFR C677T* within big tumor size, advanced tumor stage, and involvement of cancer in lymph nodes, overexpression of HER2neu and dual negativity of ER and PR hormones. Also, there was a powerful significant correlation between (AA vs AC-CC) of *MTHFR A1298C* and *HER2neu *expression and tumor stage. 

Table 5, demonstrates that the most frequent haplotype of the two loci of *MTHFR* (rs1801133-rs1801131) was TC which was strongly associated with the aggressive clinical features of each of big tumor size, advanced stage, involvement of cancer in lymph nodes, overexpression of HER2neu and dual negativity of both ER and PR hormones giving highly significant P values (0.001)

**Table 1:** Clinico Pathological Data of Patients with Breast Cancer (BC).

Parameters	Breast cancer patients (n=100)(%)
Age (years)	
≤ 45	37 (37)
>45	63 (63)
Family history	
Familiar	34 (34)
Sporadic	66 (66)
Menopausal status	
Postmenopausal	60 (60)
premenopausal	40(40)
Pathology	
IDC	80 (80)
ILC	20 (20)
Estrogen receptors	
Positive	46 (46)
Negative	54 (54)
Progesterone receptors	
Positive	42 (42)
Negative	58 (58)
HER 2 neu	
Positive	69 (69)
Negative	31 (31)
Tumor size	
T1	19 (19)
T2	39 (39)
T3	23 (23)
T4	19 (19)
Lymph nodes	
N0	34 (34)
N1	37 (37)
N2	27 (27)
N3	2 (2)
Stage	
I	17 (17)
II	26 (26)
III	53 (53)
IV	4 (4)
Grade	
2	83(83)
3	17 (17)
Metastasis	
M0	96 (96)
M1	4 (4)

IDC, Invasive ductal carcinoma; ILC, Invasive lobular carcinoma; HER2Neu, Human epidermal growth factor 2

**Table 2:** *MTHFR C677T *and *A1298C* Genotypes & Alleles Distribution in Breast Cancer Patients and Control

Gene	Model	Genotype	Patients (n=100)(%)	Controls (n=60 )(%)	OR (95% CI)
*MTHFR C677T*	Codominant	CC	18 (18.0)	40 (66.7)	1.00 (Reference)
		CT	55 (55.0)	20 (33.3)	6.111 (2.870 - 13.014)
		TT	27 (27.0)	0 (0.0)	3.222 (2.196 - 4.729)
	Dominant	CC	18 (18.0)	40 (66.7)	1.00 (Reference)
		CT-TT	82 (82.0)	20 (33.3)	9.111 (4.345 - 19.106)
	Recessive	CC-CT	73 (73.0)	60 (100.0)	1.00 (Reference)
		TT	27 (27.0)	0 (0.0)	1.822 (1.562 - 2.125)
	Overdominant	CC-TT	45 (45.0)	40 (66.7)	1.00 (Reference)
		CT	55 (55.0)	20 (33.3)	2.444 (1.256 - 4.757)
	Allele	C	91 (45.5)	100 (83.3)	1.00 (Reference)
		T	109 (54.5)	20 (16.7)	(3.438 - 10.432)5.989
*MTHFR A1298C*	Codominant	AA	11 (11.0)	32 (53.3)	1.00 (Reference)
		AC	43 (43.0)	24 (40.0)	5.212 (2.233 - 12.167)
		CC	46 (46.0)	4 (6.7)	33.455 (9.778 - 114.467)
	Dominant	AA	11 (11.0)	32(53.3)	1.00 (Reference)
		AC-CC	89 (89.0)	28 (46.7)	9.247 (4.130 - 20.703)
	Recessive	AA-AC	54 (54.0)	56 (93.3)	1.00 (Reference)
		CC	46 (46.0)	(6.7) 4	11.926 (4.018 - 35.398)
	Overdominant	AA-CC	57 (57.0)	36 (60.0)	1.00 (Reference)
		AC	43 (43.0)	24 (40.0	1.132 (0.590 - 2.169)
	Allele	A	65 (32.5)	88 (73.3)	1.00 (Reference)
		C	135 (67.5)	32 (26.7)	5.712 (3.460 - 9.429)
Haplotypes (CombinedGenotypes) (C677T/A1298C)					
		CC/AA	7 (7)	22 (36.7)	1.00 (Reference)
		CC/AC	7 (7)	14 (23.3)	1.571 (0.453 - 5.450)
		C/CC	4 (4)	4 (6.7)	3.143 (0.618 - 15.978)
		CT/AA	4 (4)	10 (16.7)	1.257 (0.298 - 5.296)
		CT/AC	25 (25)	10 (16.7)	7.857 (2.556 - 24.154)
		CT/CC	26 (26)	0 (0)	4.143 (2.173 - 7.898)
		TT/AA	0 (0)	0 (0)	----------
		TT/AC	11 (11)	0 (0)	4.143 (2.173 - 7.898)
		TT/TT	16 (16)	0 (0)	4.143 (2.173 - 7.898)

OR, odds ratio; CI, confidence interval

**Table 3:** Univariate Analysis of *MTHFR C677T *and *MTHFR*

Parameters	MTHFR C677T	MTHFR C677T	OR (95% CI)	MTHFR A1298C	MTHFR A1298C	OR (95% CI)
	CT-TT	CC		AC-CC	AA	
	n (%)	n (%)		n (%)	n (%)	
Age						
45≥	(34.1)28	(50) 9	(Reference)1.00	(37.1)33	(36.4) 4	(Reference)1.00
45<	(65.9) 54	(50) 9	1.929 (0.688 - 5.405)	(62.9)56	(63.6) 7	0.970 (0.264 - 3.564)
ER/PR						
both –ve	(54.9) 45	(16.7) 3	(Reference)1.00	(51.7)46	2 (18.2)	(Reference)1.00
both +ve	21 (25.6)	15 (83.3)	0.093 (0.024 - 0.358)	(31.5)28	8 (72.7)	0.152 (0.030 - 0.768)
HER2neu						
ve-	16 (19.5)	15 (83.3)	(Reference)1.00	(25.8)23	8 (72.7)	(Reference)1.00
ve+	66 (80.5)	3 (16.7)	20.625 (5.322 - 79.924)	(74.2)66	3 (27.3)	7.652 (1.870 - 31.318)
Tumor size						
T1	(8.5) 7	(66.7) 12	(Reference)1.00	(12.4) 11	8 (72.7)	(Reference)1.00
T2-T4	(91.5)75	(33.3) 6	21.429 (6.145 - 74.729)	(87.6) 78	3 (27.3)	18.909 (4.350 - 82.191)
Lymph nodes						
N0	(23.2) 19	15 (83.3)	1.00 (Reference)	(30.3) 27	(63.6) 7	1.00 (Reference)
N1-N3	63 (76.8)	3 (16.7)	16.579 (4.334 - 63.414)	(69.7) 62	4 (36.4)	4.019 (1.085 - 14.878)
Metastasis						
M0	78 (95.1)	18 (100)	1.00 (Reference)	(95.5) 85	11 (100)	1.00 (Reference)
M1	4 (4.9)	(0) 0	1.231 (1.118 - 1.355)	(4.5) 4	(0) 0	1.129 (1.051 - 1.214)
Stage						
I-II	28 (34.1)	15 (83.3)	1.00 (Reference)	(39.3) 35	8 (72.7)	1.00 (Reference)
III-IV	54 (65.9)	3 (16.7)	9.643 (2.573 - 36.132)	(60.7) 54	3 (27.35)	4.114 (1.021 - 16.574)
Grade						
2	65 (79.3)	18 (100)	1.00 (Reference)	(80.9) 72	11 (100)	1.00 (Reference)
3	17 (20.7)	(0) 0	1.277 (1.140 - 1.430)	(19.1) 17	(0) 0	1.153 (1.060 - 1.254)

HER2Neu, Human epidermal growth factor 2; ER, estrogen receptor; PR, progesterone receptor; OR, Odds ratio; CI, confidence interval

**Table 4:** Multivariate Analysis of *MTHFR* C677T & *MTHFR* A1298C

Parameters	*MTHFR C677T *(CC vs CT-TT)			(*MTHFR A1298C *(AA vs AC-CC		
	OR	95% C.I.		OR	95% C.I.	
		Lower limit	Upper limit		Lower limit	Upper limit
HER 2 Neuo ( -ve, +ve)	20.625	5.322	79.924	7.652	1.870	31.318
Tumor Size (T1, T2-T4)	21.429	6.145	74.729	18.909	4.350	82.191
LYMPH Nodes (N0, N1-N3)	16.579	4.334	63.414	4.019	1.085	14.878
(M0,M1)Metastasis	3.73E+08	0	.	2.09E+08	0.000	.
Stage (I-II, III-IV)	9.643	2.573	36.132	4.114	1.021	16.574
Grade (2, 3)	4.47E+08	0	.	2.47E+08	0.000	.
ER /PR (Both -ve, Both +ve)	0.806	0.713	0.911	0.843	0.727	0.976

HER2Neu, Human epidermal growth factor 2; ER, estrogen receptor; PR, progesterone receptor; OR, Odds ratio; CI, confidence interval

**Table 5:** Association of MTHFR Haplotypes with Clinical Characteristics of Breast Cancer PatientS

Haplotype (C677T -A1298C)	n (%)		OR (95% CI)
	45<	45 =>	
C-A	56 (32.2)	72 (49.3)	1.00 (Reference)
C-C	39 (22.4)	24 (16.4)	2.089(1.127-3.872)
T-A	17 (9.8)	8 (5.5)	2.732(1.100-6.788)
T-C	62 (35.6)	42 (28.8)	1.898 (1.123 - 3.208)
	PR		
	ve+	ve-	
C-A	32 (38.1)	18 (15.5)	1.00 (Reference)
C-C	21 (25.0)	20 (17.2)	0.591 (0.255 - 1.370)
T-A	5 (6.0)	10 (8.6)	0.281 (0.083 - 0.952)
T-C	26 (31.0)	68 (58.6)	0.215 (0.103 - 0.448)
	ER		
	ve+	ve-	
C-A	34 (37.0)	16 (14.8)	1.00 (Reference)
C-C	22 (23.9)	19 (17.6)	0.545 (0.232 - 1.280)
T-A	7 (7.6)	8 (7.4)	0.412 (0.127 - 1.334)
T-C	29 (31.5)	65 (60.2)	0.210 (0.100 - 0.439)
	HER 2 Neuo		
	ve+	ve-	
C-A	23 (16.7)	27 (43.5)	1.00 (Reference)
C-C	25 (18.1)	16 (25.8)	1.834 (0.793 - 4.242)
T-A	9 (6.5)	6 (9.7)	1.761 (0.545 - 5.692)
T-C	81 (58.7)	13 (21.0)	7.314 (3.262 - 16.403)
	Tumor Size		
	T2 - T4	T1	
C-A	32 (19.8)	18 (47.4)	1.00 (Reference)
C-C	29 (17.9)	12 (31.6)	1.359 (0.560 - 3.299)
T-A	12 (7.4)	3 (7.9)	2.250 (0.560 - 9.040)
T-C	89 (54.9)	5 (13.2)	10.012 (3.434 - 29.190)
	Lymph Nodes		
	N1 - N3	N0	
C-A	25 (18.9)	25 (36.8)	1.00 (Reference)
C-C	20 (15.2)	21 (30.9)	0.952 (0.417 - 2.175)
T-A	10 (7.6)	5 (7.4)	2.000 (0.597 - 6.695)
T-C	77 (58.3)	17 (25.0)	4.529 (2.111 - 9.721)
	Stage		
	III – IV	I – II	
C-A	17 (14.9)	33 (38.4)	1.00 (Reference)
C-C	19 (16.7)	22 (25.6)	1.676 (0.718 - 3.915)
T-A	10 (8.8)	5 (5.8)	3.882 (1.143 - 13.185)
T-C	68 (59.6)	26 (30.2)	5.077 (2.424 - 10.634)
	Grade		
	3	2	
C-A	2 (5.9)	48 (28.9)	1.00 (Reference)
C-C	9 (26.5)	32 (19.3)	6.750 (1.368 - 33.304)
T-A	2 (5.9)	13 (7.8)	3.692 (0.474 - 28.783)
T-C	21 (61.8)	73 (44.0)	6.904 (1.548 - 30.801)
	Metastasis		
	M1	M0	
C-A	0 (0.0)	50 (26.0)	1.00 (Reference)
C-C	2 (25.0)	39 (20.3)	0.951 (0.888 - 1.019)
T-A	1 (12.5)	14 (7.3)	0.933 (0.815 - 1.069)
T-C	5 (62.5)	89 (46.4)	0.947 (0.903 - 0.993)

HER2Neu, Human epidermal growth factor 2; ER, estrogen receptor; PR, progesterone receptor; OR, Odds ratio; CI, confidence interval

## Discussion

Breast cancer is a complex and heterogeneous disease and the main cause of cancer death in females (Castigilia et al., 2019). Various environmental and genetic factors, with mutations in more than one polymorphism (*SNPs*) play interrelated roles in the pathophysiology of cancer, disease progression, and spontaneous or drug-induced viral clearance (Omran and Ibrahim et al., 2013; Moghaddam et al., 2018; Bagheri-Hossein et al., 2020). Therefore, the present study mainly aims to investigate the role of two functional and signalized SNPs in the MTHFR gene, C677T (rs 1801133) and A1298C (rs 1801131) as risk factors for breast cancer patients. These mutant alleles of MTFHR polymorphic variants have been associated with the decreased Folate enzyme activity, as its metabolism works as a Cycle; interact with each other through their substrates, causing increased levels of plasma homocysteine (Rozen, 1997). This decreases the efficiency of purines and pyrimidine synthesis as well as DNA regulation and integrity that may contribute to malignant transformation (Duthie, 1999; Choi and Mason, 2000). Thus, this study was conducted to highlight the role of these mutant alleles that contribute in increasing cancer risk in Egyptian females’ patients by developing more aggressive phenotypes that increase tumor size, stage and spread into the nearby lymph nodes and others. 

In the analysis of the demographic and clinical features of BC patients, our data showed that the risk of BC increased by the increasing age as 63%of patients are over 45 years old. These results are in agreement with (El- Bolkainy et al., 2005; Extermann et al., 2012) who reported that by aging, DNA repair becomes less efficient, allowing more mutations, chromosomal breaks and translocations. This leads to the inactivation of tumor suppressor genes, and hinder the function of DNA repair genes. Furthermore, it was debated that family history is extremely a major influence associated with increased risk of BC. The inherited susceptibility of BC is partially attributed to the aberrant expression of DNA damage with mutations of related genes such as BRCA1 and BRCA2 (Brewer et al., 2017; Diotaiuti et al., 2020). On the other side, our findings showed that 66% of patients are sporadic and that is in accordance to the work of (Shaker and Senousy, 2019) who also found no association of BC with family history. Also, in the present study, it was revealed that 60% of BC cases were postmenopausal which is in accordance with (Kuller, 1995; Said et al., 2012; Floris et al., 2020). They explained that fat cells in patients’ breasts tend to produce greater amounts of aromatase enzyme which promote estrogen production that plays a role in both the development and growth of breast cancer.

It was stated that the single nucleotide polymorphisms (*SNPs*) are the most common form of genetic variation with more than nine million reported in public databases, causing alterations in protein structure, function and can be useful as physical markers for progression of the disease or drug response (Ibrahim et al., 2016; Alkasaby et al., 2020). Further research in molecular aspects of cancers has led to pay closer attention to study polymorphisms of genes involved in tumor genesis (Hosseini, 2013; Youssef et al., 2013; Liu et al, 2020). Findings from this present study identified that MTHFR C677T and *A1298C* polymorphisms are associated with BC. Strong correlations between Egyptian BC patients and controls are found in four genetic models including codominant, dominant, recessive and overdominant models with mostly highly significant P value (P=0.001) between MTHFR C677T genotypes (C/T +T/T) with 55% in patients vs. 33.3% in controls. Combined genotypes of MTHFR A1289C (AC+CC) percentage was much higher in patients (89%) than in controls (46.7%) with high significant difference (p=0.001). In agreement to that, it is reported that mutation of MTHFR from 677C to T and A1298 to C reduces the enzyme activity that leads to decreasing the yield of methyl donor SAM which eventually affects the methylation of DNA causing hypomethylation and consecutive activation of carcinogenic oncogenes (Weisberg et al., 1998; Liu et al., 2012; Nie et al., 2020). 

Furthermore, in this study, the frequency of T allele of MTHFR C677T in patients was 54.5% vs.16.7% in controls with highly significant value (P= 0.001), indicating that the risk of breast cancer increases by 5.98 times in individual carrying the T allele. These results are supported by previous studies conducted on different populations as Indian and Moroccan suggesting that this polymorphism could be a therapeutic target for breast cancer (Waseem et al., 2016; Hardi et al., 2018; Rahimi et al., 2019). On the other hand, some previous case-control studies of: Mexican, north Indian, Turkish and Chinese population did not report any relation (Mir et al., 2008; Ramos-Silivia et al., 2015; Kaya et al., 2016; Song et al., 2016). The reason for this discrepancy may be due to ethnic differences, different life style and gene-environmental interaction. 

Additionally, our results revealed that C allele of MTHFR A1289C is highly distributed among BC patients than controls (67.5% vs. 26.7%) giving a very high significant value (P= 0.001). These results are supported by previous researches (Hosseini et al., 2011; Liu et al., 2016). Studies conducted on Turkish and Italian population demonstrated that the risk of breast cancer increase due to the presence of C allele as well as it is attributed to decreasing the enzyme activity by15% and 30% for heterozygous (AC) and homozygous (CC) carriers respectively, as a result of change of C-terminal regulatory domain of the protein that is linked with carcinogenesis (Weisberg et al., 1998; Ergul et al., 2003; Pepe et al., 2006). However, other findings and case-control studies did not reveal any correlations (Jiao and Li, 2013; Rai et al., 2014; Awwad et al., 2015; Nie et al., 2020). 

Furthermore, the interaction between genetic polymorphisms at the two loci was judged by evaluating the combined genotypes’ effects (haplotype analysis). The genotypes of adjacent SNPs are in linkage disequilibrium and are frequently highly correlated, resulting in the major determining factors of disease predisposition compared to the single polymorphisms (Zintzaras and Lau, 2008). Thus, we analyzed the haplotype frequencies of the two MTHFR SNPs, 677C>T, and 1298A>C for BC cases comparing them with their aggressive clinical features. Our results revealed that the haplotype CT/AC was associated with elevated risk of BC (OR=7.857, 95% CI 2.556 - 24.154, P=0.001) followed by CT/CC, TT/AC and TT/CC haplotypes (OR=4.143, 95% CI 2.173 - 7.898, P=0.001). This indicates that the presence of the mutant haplotype generated less active thermo labile enzymes leading to global hypomethylation, increasing the risk of chromosomal breaks and consequently more aggressive features of breast carcinogenesis. These findings are in agreement with studies done by (Gao et al., 2009; Zhong et al., 2014). On the contrary, other studies among women from different populations reported no associations (Chou et al., 2006; Floris et al., 2020). Meanwhile, similar studies showed inconclusive results as, the C-C haplotype was protective in East Asian and German populations (Justenhoven et al., 2005; Huang et al., 2011), while the C-A was protective among the South-Eastern European women (Papandreou, 2012).The T-C ,T-A haplotype in Jordanian and Caucasians populations were more susceptible to develop BC (Zhong et al., 2014; Awwad et al., 2015). 

In this study, we performed further analysis of Univariate, Multivariate and Haplotype to investigate the relationship between the clinicopathological parameters and the distributions of *SNPs* of *MTHFR C677T* and A1289C genotypes in BC Egyptian patients. Our promising results of associations are represented by the significant correlations of (CT+TT) of *MTHFR C677T *and (AC+CC) of *MTHFR A1289C* polymorphisms by developing more aggressive clinical features. Our findings of haplotype analysis among Egyptian females also clarified that the most frequent haplotype of the two loci of* MTHFR* (rs1801133-rs1801131) is TC which was significantly associated with dual negativity of both ER and PR hormones, overexpression of *HER2neu*, lymph node metastasis and tumor size. These findings are evidenced by earlier reports (Rezende et al., 2017; Castigilia et al., 2019). However, there was no correlation of our data with age or disease onset. Other studies on Moroccan and Indian cases reported no statistical significant association with clinicopathological characteristics (Waseem et al., 2016; Hardi et al., 2018; Karimian et al., 2020). Moreover, all our data were confirmed in multivariate analysis to improve the validity of the results and to identify the independent association between different genotypes of the *MTHFR C677T *(rs1801133) and *MTHFR* A1289C rs1801131 and BC. The results demonstrated that both polymorphisms are correlated with BC and it can be seen that there is a tendency towards the association between aggressiveness of tumor type and (ER-/PR-), over expression of HER2 neu, tumor size and stage with the mutant allele variants of *MTHFR* rs1801133 TT and *MTHFR* rs1801131 CC giving very highly significant values. 

To sum up, the current study revealed clear evidence of the strong significant association of the genetic polymorphisms mutations of *MTHFR* at *C677T* and *A1289C* with the increasing risk of BC among Egyptian females, confirmed by the Univariate, Multivariate and Haplotype analysis. Moreover, this is an early study demonstrating the correlations between *MTHFR* gene polymorphisms and the pathological and clinical features. This helps in providing a molecular detection by using these polymorphisms as predictive and prognostic markers for the development of breast cancer disease.
